# Two Engineered *Bacillus subtilis* Surfactin High-Producers: Effects of Culture Medium, and Potential Agricultural and Petrochemical Applications

**DOI:** 10.3390/biology15020146

**Published:** 2026-01-14

**Authors:** Graciely Gomes Corrêa, Elvio Henrique Benatto Perino, Cristiano José de Andrade, Maliheh Vahidinasab, Lucas Degang, Behnoush Hosseini, Lars Lilge, Vitória Fernanda Bertolazzi Zocca, Jens Pfannstiel, Danielle Biscaro Pedrolli, Rudolf Hausmann, Jonas Contiero

**Affiliations:** 1 Industrial Microbiology Laboratory, Department of General and Applied Biology, Institute of Biosciences, São Paulo State University (UNESP), Rio Claro 13506-900, SP, Brazil; jonas.contiero@unesp.br; 2 Associate Laboratory of IPBEN Prof. Dr. Alcides Serzedello, Institute for Research in Bioenergy—IPBEN, Rio Claro 13506-900, SP, Brazil; 3 Department of Bioprocess Engineering (150k), Institute of Food Science and Biotechnology, University of Hohenheim, 70599 Stuttgart, Germany; m.vahidinasab@tum.de (M.V.); l.lilge@mul-ct.de (L.L.); rudolf.hausmann@uni-hohenheim.de (R.H.); 4 Department of Chemical Engineering and Food Engineering, Technological Center, Federal University of Santa Catarina, Florianópolis 88040-900, SC, Brazil; cristiano.andrade@ufsc.br (C.J.d.A.); lucasdegang19@gmail.com (L.D.); 5 Department of Phytopathology (360a), Institute of Phytomedicine, Faculty of Agricultural Sciences, University of Hohenheim, 70599 Stuttgart, Germany; behnoush.hosseini@uni-hohenheim.de; 6 Department of Youth Development, Medical University Lausitz—Carl Thiem, 03048 Cottbus, Germany; 7 Synthetic Biology Laboratory, Department of Bioprocess Engineering and Biotechnology, School of Pharmaceutical Sciences, São Paulo State University (UNESP), Araraquara 14800-903, SP, Brazil; vitoria.zocca@unesp.br (V.F.B.Z.); danielle.pedrolli@unesp.br (D.B.P.); 8 Core Facility Hohenheim, Mass Spectrometry Unit, University of Hohenheim, 70599 Stuttgart, Germany; jens.pfannstiel@uni-hohenheim.de

**Keywords:** biosurfactant, antifungal activity, oil degradation test, biotechnology, synthetic biology, bioeconomy

## Abstract

Two genetically engineered *Bacillus subtilis* strains, BMV9 and BsB6, were evaluated in terms of culture medium (effect of nutrients on surfactin yield) and potential biotechnological applications of surfactin in agriculture and the petrochemical industry. BMV9 (*spo0A3*; abrB*; Δ*manPA*; *sfp+*) is, to date, the highest surfactin producer reported scientifically, and BsB6 is a *sfp*+ laboratory derivative strain that has also demonstrated considerable production potential. To assess their performance, fermentation experiments were conducted in shake flasks using two different culture media, a mineral salt medium and a complex medium, each supplemented with 2% (*w*/*v*) glucose. Lipopeptides (surfactin and fengycin) were extracted and quantified at multiple time points (up to 48 h) via high-performance thin-layer chromatography (HPTLC). Optical density, residual glucose, and pH were monitored throughout the cultivation. In parallel, microbial growth in both media were also validated in small-scale cultivation approaches. Antifungal activity of culture supernatants and lipopeptide extracts was tested against two *Diaporthe* species, key phytopathogens in soybean crops. Given the agricultural relevance of these pathogens, the biocontrol potential of lipopeptides represents a sustainable alternative to conventional chemical fungicides. Additionally, oil displacement tests were performed to evaluate the efficacy of surfactin in enhanced oil recovery (EOR), bioremediation, and related petrochemical processes. High-resolution LC-MS/MS analysis enabled structural characterization and relative quantification of the lipopeptides. Overall, these investigations provide a comprehensive comparison of strain production performance and the associated impact of cultivation media, aiming to define the optimal conditions for economically viable surfactin production and to explore its broader biotechnological applications in agriculture and the petrochemical industry.

## Simple Summary

This study explores ecological solutions for agriculture and the petroleum industry. The goal was to evaluate the potential of two strains of bacteria, named *Bacillus subtilis*, which were genetically modified to produce a natural soap-like molecules known as surfactin. We evaluated the surfactin production in different types of nutrients, named culture media, and then explored two practical applications. First, we checked if surfactin could work as a biological control, meaning a natural pesticide, against two types of fungi that cause diseases in soybean crops. Second, we investigated the effectiveness of surfactin in helping to clean up oil spills or improve oil extraction in industrial processes. As a result, the produced surfactin proved to be effective both against the soybean fungi and in separating oil and water. In conclusion, this research defines the ideal cultivation conditions to produce large quantities of surfactin in an economically viable way. This work is valuable to society, since it offers a sustainable alternative to traditional agricultural chemicals and contributes to the development of cleaner and more efficient methods for oil recovery and environmental cleanup.

## 1. Introduction

Biosurfactants are amphiphilic compounds produced by microorganisms, characterized by their surface-active properties, which enable a wide range of industrial applications, including bioremediation, agriculture, and pharmaceuticals [1]. Among these, surfactin and fengycin, produced by *Bacillus subtilis*, have attracted significant attention due to their potent antimicrobial, antifungal, and emulsifying activities [2,3,4,5]. These biosurfactants also hold promise in various petrochemical processes such as enhanced oil recovery (EOR), bioremediation, produced water treatment, and mitigation of oil sheens [6]. Surfactin, in particular, is a cyclic lipopeptide with exceptional surface activity, making it a strong candidate for applications in oil recovery, food processing, and pharmaceutical formulations [7]. Similarly, fengycin is well known for its antifungal properties, offering potential for environmentally friendly strategies in agricultural disease control [8].

Currently, most surfactants are produced using petroleum-derived substrates, and the replacement of synthetic surfactants by biosurfactants as a more sustainable and environmentally friendly alternative still faces several limitations [9,10,11]. Biosurfactants can be significantly more expensive than synthetic surfactants, mainly due to the yield of production, and the low productivity of natural microbial strains, which are unable to produce sufficient amounts for economically viable processes. Additionally, excessive foaming during cultivation, due to the aeration and agitation required in bioreactors, and the complexity of downstream purification steps further hinder large-scale applications. Therefore, improving upstream processes, particularly through the development and use of genetically engineered microorganisms, is essential to enhance biosurfactant yields and enable their broader implementation in a bioeconomy context [12,13].

*B. subtilis* has emerged as a promising host for improving microbial biosurfactant production due to its advantageous physiological and genetic characteristics. As a Gram-positive bacterium with Generally Recognized As Safe (GRAS) status, *B. subtilis* offers a safe platform for industrial applications. Its genetic tractability, rapid growth, and well-characterized regulatory networks make it highly amenable to metabolic engineering and synthetic biology approaches [14,15]. Among the biosurfactants of interest, surfactin, a powerful lipopeptide with surface-active and antimicrobial properties, stands out for its potential applications in agriculture, bioremediation, and pharmaceuticals. However, many laboratory strains of *B. subtilis* do not naturally produce surfactin due to mutations or deletions in the *sfp* gene, which encodes a 4′-phosphopantetheinyl transferase essential for activating non-ribosomal peptide synthetases involved in surfactin biosynthesis. Therefore, the reintroduction or activation of a functional *sfp* gene (*sfp+*) is a key genetic modification required to enable and enhance surfactin production in engineered strains of *B. subtilis* [16,17,18,19].

The genetic manipulation of *B. subtilis* has thus become a central strategy in efforts to increase biosurfactant yields. One of the most prominent examples is the BMV9 strain [19], derived from the high-producing *B. subtilis* 3NA [20]. BMV9 is recognized as one of the most efficient surfactin producers described to date [21], with demonstrated capacity to utilize low-cost and sustainable carbon sources such as vegetable juice for economically feasible production [22]. This strain harbors multiple targeted genetic modifications, including a frameshift mutation in the *spo0A* gene locus (*spo0A3*) that confers a sporulation-deficient phenotype advantageous for continuous fermentation processes. The restoration of the *sfp* gene (*sfp+*) enables lipopeptide biosynthesis, while a mutation in the *abrB* gene extends the regulatory protein from 96 to 107 amino acids (abrB*), potentially altering key transcriptional networks associated with secondary metabolism [20,23].

The efficiency of biosurfactant production is also highly dependent on environmental factors, including nutrient composition and carbon-to-nitrogen (C/N) ratio [24,25]. To better understand how media composition influences lipopeptide synthesis, this study compares BMV9 and BsB6 in two different culture media: Mineral Salt Medium (MSM), a defined minimal medium, and Power Medium (PW), a complex medium rich in nutrients [26,27].

Furthermore, this work aims to (i) compare the growth kinetics and biosurfactant production of the *B. subtilis* strains BMV9 and BsB6 in different culture media; (ii) assess the impact of medium composition on surfactin and fengycin yields; (iii) evaluate the antifungal activity of the produced biosurfactants; and (iv) explore their potential applications in the petrochemical sector. The findings will contribute to the optimization of *B. subtilis* strains for industrial biosurfactant production and expand their application potential in biotechnology and agriculture. Moreover, the comparative evaluation of both strains under distinct cultivation conditions provides valuable insights into how genetic background and medium composition influence the microbial conversion of renewable bioresources into value-added bioproducts, such as surfactin contributing to the advancement of sustainable bioprocesses.

## 2. Material and Methods

### 2.1. Bacterial Strains, Culture Media, and Chemicals

The strains used in this study were two genetically modified surfactin producers: *B. subtilis* BMV9 [19] and BsB6 (unpublished). They were developed at the Department of Bioprocess Engineering (University of Hohenheim, Stuttgart/Germany), and at the Department of Bioprocess Engineering and Biotechnology (UNESP, São Paulo/Brazil), respectively.

Cultivation experiments were performed using two different culture media. (i) Modified Mineral Salt Medium (MSM) [26] composed of following solutions: buffer + nitrogen source (7.12 g/L Na_2_HPO_4_·2H_2_O, 4.08 g/L KH_2_PO_4_, 6.61 g/L (NH_4_)_2_SO_4_), magnesium sulfate (0.20 g/L MgSO_4_·7H_2_O), sugar source (glucose (C_6_H_12_O_6_)), trace elements (1.49 mg/L Na_2_EDTA·2H_2_O, 0.78 mg/L CaCl_2_, 1.11 mg/L FeSO_4_·7H_2_O, and 0.17 mg/L MnSO_4_·H_2_O); (ii) a complex medium (Power—PW) [27], composed of solutions: buffer + nitrogen source (1 g/L yeast extract, 25 g/L NaNO_3_, 0.33 g/L, KH_2_PO_4_, 0.5 g/L Na_2_HPO_4_·2H_2_O), magnesium sulfate (0.15 g/L MgSO_4_·H_2_O), sugar source (glucose (C_6_H_12_O_6_)), trace elements (7.5 mg/L, CaCl_2_, 6 mg/L MnSO_4_·H_2_O, 6 mg/L FeSO_4_·7H_2_O). The pH of the buffer + nitrogen source solution was adjusted to 7.0. The buffer + nitrogen source, and the magnesium sulfate solutions were sterilized at 121 °C for 15 min. To prevent Maillard reactions, glucose was sterilized separately by autoclaving and supplemented to the cultures at a final concentration of 20 g/L (2%, *w*/*v*) in both media. The trace elements solution was sterilized by filtration through a 0.22 µm hydrophilic membrane and stored in an amber bottle to protect it from light.

All chemicals were obtained from Carl Roth GmbH + Co. KG (Karlsruhe, Germany). Standards of surfactin and fengycin for quantitative measurements were acquired from Sigma-Aldrich (St. Louis, MO, USA) and Lipofabrik (Lesquin, France), respectively. Commercial surfactin (sodium surfactin) was purchased from Kaneka Corporation (Osaka, Japan).

### 2.2. Shake Flask Cultivation

The influence of different media on both strains, BMV9 and BsB6, were assessed through shake flask cultures. For pre-culture preparation, 10 µL of glycerol stock culture was inoculated into 10 mL of Luria–Bertani (LB) broth (10 g/L tryptone, 5 g/L yeast extract, 5 g/L NaCl) in baffled flasks, followed by overnight incubation at 37 °C and 120 rpm. Afterwards, the bacterial suspension was used to inoculate each medium to an optical density (OD_600_) of 0.1. The main cultivations were conducted in 500 mL baffled flasks with a working volume of 50 mL, maintained at 37 °C and 120 rpm for 48 h in an incubation shaker (Innova 44^®^R, Eppendorf AG, Hamburg, Germany). Samples were collected at 0, 10, 18, 24, 28, 32, and 48 h after inoculation (hai). All shake flask experiments were performed in biological triplicates.

### 2.3. Small-Scale Cultivation Approaches

Both strains of *B. subtilis* were cultivated in transparent 96-well plates with a starting OD_600_ of 0.1 in a final volume of 100 µL. Each culture was prepared in biological triplicates and incubated for 12 h at 37 °C with orbital shaking at 200 rpm (3 mm amplitude; ≈3.3 Hz) using a microplate reader (FLUOstar Omega version 5.70 R2, BMG LABTECH GmbH, Ortenberg, Germany). Measurements of OD_600_ were monitored every 10 min for each well.

### 2.4. Assessment of Growth, Glucose Consumption, pH Variations, and Lipopeptide Quantification

The absorbance of all collected samples in the shake flask cultivations was measured using a cell density meter CO8000 (Biochrom Ltd., WPA Biowave, Cambridge, UK) set to a wavelength of 600 nm (OD_600_).

To remove biomass, cultivation samples were centrifuged at 3890× *g* for 10 min at 4 °C using a Multifuge X3R (Thermo Fisher Scientific, Waltham, MA, USA). The resulting supernatants were analyzed for glucose concentration using high-performance thin-layer chromatography (HPTLC) (CAMAG AG, Muttenz, Switzerland), following the protocol previously described [28]. Briefly, lipopeptides were extracted from the supernatants three times using a chloroform-methanol mixture (2:1, *v*/*v*), and the combined organic phases evaporated under vacuum at 45 °C and 10 mbar for 1 h. The residue was dissolved in methanol to restore the sample volume. The separation was carried out using a mobile phase composed of chloroform, methanol, and water (65:25:4, *v*/*v*/*v*) over a migration distance of 60 mm. The plates were then scanned at 195 nm, and surfactin concentrations were determined by comparing the peak areas to those of standard solutions. For fengycin, besides the first development, a second development in butanol/ethanol/0.1% acetic acid (1:4:1, *v*/*v*/*v*) was used. The separation was performed with acetonitrile and water (85:15, *v*/*v*) as the mobile phase, allowing a migration distance of 70 mm. For post-separation, the plates were treated with a diphenylamine (DPA) reagent, which was prepared by dissolving 2.4 g diphenylamine and 2.4 g aniline in 200 mL methanol, followed by the addition of 20 mL 85% phosphoric acid. The plates were scanned at 620 nm, and glucose levels were calculated using a standard calibration curve. The pH was measured using pH indicator strips (range pH 0–14; Merck, Darmstadt, Germany).

#### Data Analysis

The product yield per biomass (Y_P/X_) [Equation (1)] and product yield per substrate (Y_P/S_) [Equation (2)] were calculated at the maximum concentrations of surfactin. The biomass yield per substrate (Y_X/S_) [Equation (3)] was determined at the maximum cell dry weight (CDW), with a correction factor of 4.31 [22] applied for CDW calculation. The specific productivity (q_P/X_) [Equation (4)] was calculated by dividing Y_P/X_ by the fermentation time (time at maximum lipopeptide concentration—time at initial lipopeptide concentration). The specific growth rate was calculated at highest CDW time point [Equation (5)]. All values were adjusted by the volume at the time point of the sample collection.

(1) $$Y_{P/X}=\frac{m_{lip\;max}-m_{lipt0}}{m_{{CDW}_{lip\;max}}-m_{{CDW}_{t0}}}$$

(2) $$Y_{P/S}=\frac{m_{lip\;max}-m_{lip\;t0}}{\Delta S}$$

(3) $$Y_{X/S}=\frac{m_{{CDW}_{max}}-m_{{CDW}_{t0}}}{\Delta S}$$

(4) $$q_{P/X}=\frac{Y_{P/X}}{\Delta t}$$

(5) $$\mu =\frac{ln\frac{m_{{CDW}_{max}}}{m_{{CDW}_{t0}}}}{t_{{CDW}_{max}}-t_{{CDW}_{t0}}}$$

where m: mass of lipopeptide (surfactin or fengycin); CDW: cell dry weight; ΔS: substrate consumption and t: period of time. The statistical analyses were conducted using SigmaPlot software (version 13).

### 2.5. LC-MS/MS Analysis and Relative Quantification of Lipopeptides

Lipopeptides were extracted from culture supernatants collected after 24 h of fermentation, as previously described [22]. The extracts were analyzed by liquid chromatography coupled with tandem mass spectrometry (LC-MS/MS) for identification and relative quantification of surfactin and fengycin isoforms.

Analyses were performed using an Ultra-High Performance Liquid Chromatography (UHPLC) system (Agilent, Waldbronn, Germany) coupled to a Q-Exactive Plus Orbitrap mass spectrometer with a heated electrospray ionization (HESI) source (Bruker Daltoniks, Bremen, Germany). Separation of lipopeptides was achieved using an ACQUITY CSH C18 column (1.7 µm, 2.1 × 150 mm, Waters, Eschborn, Germany), maintained at 40 °C. Samples were dissolved in methanol, and 10 µL was injected.

The mobile phases consisted of 0.2% (*v*/*v*) formic acid in water (A) and 0.2% (*v*/*v*) formic acid in acetonitrile (B). The flow rate was 0.3 mL/min, with the following gradient: 40–70% B (0–12 min), 70–95% B (12–20 min), held at 95% B (20–24 min), and re-equilibrated to 40% B (24–26 min).

The HESI source operated in positive ion mode with a spray voltage of 4.20 kV, capillary temperature of 360 °C, auxiliary and sweep gas pressure of 20 and 60 (arbitrary units), respectively, auxiliary gas heater at 150 °C, and an S-Lens RF level of 50%. External calibration was performed using positive mode calibration solution (Pierce™, Thermo Fisher Scientific, Rockford, IL, USA).

Mass spectra were acquired in the 500–1600 *m*/*z* range at 70,000 FWHM resolution, with an AGC target of 1.0 × 10^6^ and maximum injection time of 100 ms. Data-dependent MS/MS spectra were recorded for the five most intense precursor ions, using a resolution of 17,500 FWHM, AGC target of 3.0 × 10^6^, 100 ms injection time, and stepped collision energies of 15, 30, and 45 eV. An inclusion list containing the *m*/*z* values of known surfactin and fengycin isoforms was applied to ensure targeted fragmentation.

Lipopeptides were identified based on accurate precursor *m*/*z* values, chromatographic retention times, and characteristic fragment ions in MS/MS spectra. In cases where fragmentation patterns were not conclusive, tentative assignments were made using retention times and reference data from literature [29,30,31].

Relative quantification was performed by comparing peak areas from extracted ion chromatograms (XICs) of precursor ions across samples. All analyses were performed in triplicates.

### 2.6. Antifungal Activity

#### 2.6.1. Samples Preparation

In the first assay, a 1 mL aliquot of the 24 h supernatant obtained from the fermentation of *B. subtilis* strains in this study was utilized. For the second assay, a 28 h lipopeptide extract was subjected to rotary evaporation and then resuspended in 1 mL of sterile distilled water. Each sample was individually filtered using a 0.22 µm PES membrane. Sterile distilled water, processed in the same manner, served as the negative control.

#### 2.6.2. Fungal Pathogens

The fungal pathogens *Diaporthe longicolla* and *D*. *caulivora* (isolates DPC_HOH20 and DPC_HOH2, respectively), which were isolated from European soybean seeds [32], were used in this study to evaluate antifungal activity. *D. longicolla* was utilized in both experiments, while *D. caulivora* was included only in the second.

#### 2.6.3. Antifungal Assay

Antifungal activity of the biosurfactant was evaluated according to a methodology adapted from a previously published work [33]. The Petri dishes were prepared with a medium composed of Potato Dextrose Agar (PDA; Sigma-Aldrich, St. Louis, MO, USA), and LB agar in a 1:1 ratio. A 0.6 cm mycelial plug, taken from the actively growing margins of a 10-day-old fungal culture, was placed near one corner of the dish, 15 mm from the edge. On the opposite side of the plate, also 15 mm from the edge, a hole with the same diameter as the mycelial plug was made in the medium, and the agar block was removed. Into this space, 100 µL of each filtered sample and the negative control were added. All samples and negative controls were tested in triplicate. The plates were incubated at 25 ± 2 °C, protected from light, to ensure optimal growth conditions for the fungi.

Fungal growth was monitored daily. After ten days, the radius of inhibition was measured. The percentage of inhibition of radial growth (PIRG) was calculated using the following formula:

PIRG (%) = [(R1 − R2)/R1] × 100

where R1 represents the radius of the fungal colony in the control, and R2 represents the radius of the fungal colony in the presence of the bacterial biosurfactant extract.

### 2.7. Oil Displacement Test

The oil displacement test was performed with adaptations based on a previously reported work [6]. Initially, 30 mL of distilled water were added to a Petri dish with a diameter of 8 cm, followed by the application of crude oil (50 µL), obtained from a Brazilian offshore platform, forming an oil film on the water surface. Subsequently, 20 µL of the surfactant solution was applied upon the surface of the oil film. The displacement halo was observed after 30 s and subsequently measured using the ImageJ software (version 1.54g; National Institutes of Health, Bethesda, MD, USA) [34]. For this experiment, 200 mg/L solutions of samples produced via fermentation (24 h) were used. The samples were then codified according to the culture medium and the strain, respectively: *B. subtilis* strains cultivated in MSM (A—BMV9, and B—BsB6), and PW (C—BMV9, and D—BsB6) media. Finally, A, B, C, and D were used and compared with solutions of commercial surfactin Kaneka Corporation (Osaka, Japan) and sodium dodecylbenzenesulfonate (SDBS). Each experiment was conducted in triplicate. Statistical analyses were performed using Statistica software (version 13.5.0.17; TIBCO Software Inc., Palo Alto, CA, USA).

## 3. Results and Discussion

### 3.1. Culture Medium Comparison

**Figure 1:**
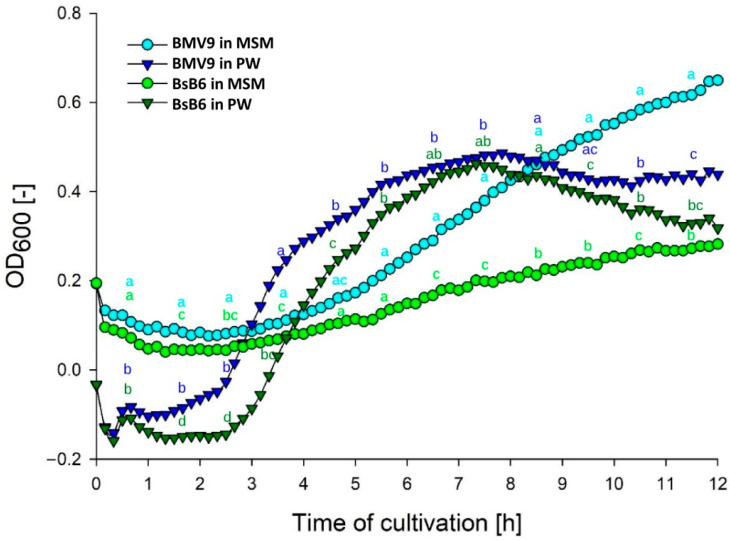
Growth curves of the *B. subtilis* strains BMV9 (blue shades) and BsB6 (green shades) cultivated in MSM (Mineral Salt Medium) (circles) and PW (Power Medium) (inverted triangles) media. Data obtained using a microplate reader. Statistical analysis was performed on the mean OD_600_ over 1 h intervals using one-way ANOVA followed by Tukey’s test. Different letters indicate significant differences at the same interval (*p* < 0.05).

Bacterial growth carried out on a small scale using a microplate reader exhibited a similar pattern to that observed when scaled up to shake flask cultures, as illustrated in Figure 1. In the MSM medium, bacterial growth was delayed suggesting microbial adaptation during the lag phase. In contrast, in the PW medium, the exponential phase was well-defined, showing a distinct growth peak followed by a pronounced cell death phase. In addition, BMV9 strain grew significantly 6 hai more in comparison to the BsB6 strain in MSM showing the impact of the non-sporulating phenotype of BMV9 strain. BMV9 also showed a significant difference in cell growth between 1 and 5 h (exponential phase) in the PW medium. Statistical analysis was performed on the mean OD_600_ over 1 h intervals using one-way ANOVA followed by Tukey’s test.

**Figure 2:**
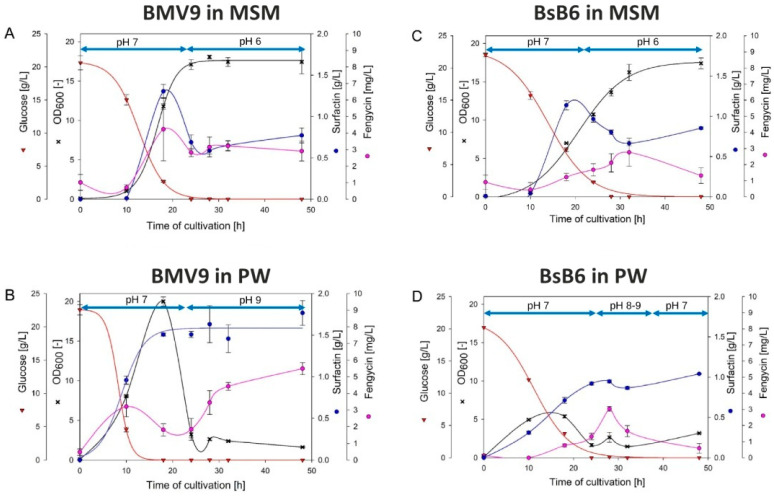
Cultivation pattern of *B. subtilis* BMV9 in MSM (Mineral Salt Medium) (**A**) and PW (Power Medium) (**B**) medium, and BsB6 strain in MSM (**C**) and PW (**D**) medium. Red inverted triangles, glucose concentration (g/L); black crosses, OD_600_; blue circles, surfactin concentration (g/L); pink circles, fengycin concentration (mg/L). Blue arrows represent the pH in the specific period. Error bars indicate the standard error of the mean (SEM).

Similarly to the microplate cultures, in shake flask cultivation (Figure 2), both strains exhibited delayed growth in MSM medium. In addition, BMV9 also showed in both experiments a maximum OD_600_ value in the complex medium, while the BsB6 strain showed maximum OD_600_ in the MSM medium. In PW medium, while both strains reached the peak of the exponential phase at 18 h, a subsequent decline in growth was observed (Figure 2).

Regarding surfactin production, the maximum production was reached at 18 hai in MSM medium (1.31 g/L and 1.14 g/L for BMV9 and BsB6 strains, respectively) and at 48 h in PW medium (1.77 g/L and 1.04 g/L for BMV9 and BsB6 strains, respectively).

For fengycin production, the highest levels were achieved in PW medium, with 5.49 mg/L (48 hai) and 3.03 mg/L (28 hai) for BMV9 and BsB6 strains, respectively.

Initial glucose was depleted after 24 h of fermentation. In MSM medium, the stationary phase was reached once glucose was consumed, and surfactin levels declined rapidly before stabilizing. On the other hand, in PW medium, glucose depletion caused a sharp slowdown in growth, although surfactin levels remained constant even after entering the stationary phase.

Analyzing the pH during fermentation in PW medium, an alkalization was observed, with the pH increasing to around 9. Conversely, the MSM medium exhibited a buffering effect, leading to acidification to a pH of approximately 6. Studies indicate that this pH range does not negatively affect surfactin stability, which remains stable between pH 5 and 9 [11]. However, maintaining a pH closer to neutral is advantageous, highlighting the importance of the buffering effect for supporting *Bacillus* growth during fermentation. In this context, MSM medium can be considered more suitable for the bioprocess.

Differences in the growth kinetics, biosurfactant yields and productivity varied depending on the strain and medium, highlighting the strain- and medium-specific impacts on biosynthetic efficiency (Table 1). Theoretical values in Table 1 were calculated for the complex medium from the amount of glucose added as a measurable carbon source. However, complex media also contain other carbon compounds that were not quantified, meaning the carbon available to microorganisms may be higher than estimated. Therefore, these theoretical values should be interpreted as indicative rather than exact.

The specific growth rate (μ) was highest for BMV9 in PW medium (0.285 h^−1^), indicating rapid metabolic activity in this nutrient-rich medium. Conversely, BsB6 in MSM exhibited the lowest μ (0.094 h^−1^), reflecting slow adaptation to minimal nutrients. Biomass yield per substrate (Y_X/S_) was similar for BMV9 in PW (0.205 g/g) and MSM (0.202 g/g), whereas BsB6 in PW had the lowest Y_X/S_ (0.078 g/g), suggesting inefficient biomass formation under complex nutrient conditions.

Surfactin production varied significantly based on the medium. BMV9 in PW reached the highest Y_P/X-surf_ (5.284 g/g) at 48 h, highlighting its superior capacity for surfactin synthesis in nutrient-rich media. However, the highest Y_P/S-surf_ (0.085 g/g) was observed for BsB6 in MSM, reflecting better substrate-to-product conversion efficiency in this minimal medium, including low biomass formation. The specific productivity (q_P/X-surf_) for surfactin was notably higher for BMV9 in PW (0.110 g/g·h), demonstrating its enhanced biosynthetic potential.

When the fengycin production was analyzed, a similar outcome was found, with BMV9 in PW reaching the highest Y_P/X-fen_ (13.67 mg/g) after 48 h. However, in MSM, BMV9 showed lower fengycin yields (Y_P/X-fen_ = 1.114 mg/g, Y_P/S-fen_ = 0.173 mg/g). The BsB6 strain exhibited its highest Y_P/X-fen_ (4.817 mg/g) in PW, though this was substantially lower than BMV9.

Additionally, surfactin and fengycin production are known to be regulated by quorum sensing (QS), which governs biosynthetic gene expression in response to cell density [35]. These discrepancies may result from genomic differences between BMV9 and the BsB6 strain, affecting biosynthetic pathways and regulatory mechanisms. BMV9 may possess a well-regulated QS system, enabling efficient fengycin production already described before [19,36]. In contrast, a weaker QS signaling or mutations in the genome of BsB6 strain could explain its reduced production yields. The specific productivity for fengycin (q_P/X-fen_) was highest for BMV9 in PW (0.285 mg/g·h), indicating that complex nutrients not only support growth but also enhance biosynthetic activity. These findings are consistent with suggestions explaining opposite acting regulatory circuits for stimulating fengycin and surfactin biosynthesis [36].

These results are also consistent with previous published data [22], which reported a surfactin Y_P/X-surf_ of 0.263 g/g and μ of 0.193 h^−1^ for BMV9 in MSM supplemented with 20 g/L glucose. While BMV9 in PW in this study substantially outperformed MSM in terms of Y_P/X-surf_ and q_P/X-surf_, the BsB6 strain demonstrated similar performance in MSM, albeit at lower overall titers. The findings of [22] also highlight the potential for fed-batch strategies to enhance lipopeptide yields, achieving 2.77 g/L surfactin in MSM in 1 L shake flasks.

Interestingly, [22] reported a higher fengycin yield (1.88 mg/g) in MSM than found in this study (0.118 mg/g for BMV9 in MSM). This discrepancy may stem from differences in fermentation monitoring times. Their work also demonstrated that fed-batch cultivation with a waste residue feeding strategy significantly enhanced surfactin yields, reaching 2.77 g/L; while [21] reached 36 g/L with glucose feeding strategy. This highlights the potential for optimizing these strains’ performances in MSM using dynamic feeding strategies.

The BMV9 strain performs optimally in PW, achieving the highest Y_P/X_ and q_P/X_ for both surfactin and fengycin. Conversely, MSM appears more suitable for the BsB6 strain, which exhibited better biosynthetic efficiency in minimal media. An adaptation of strain BsB6 in bioreactor processes already optimized for the BMV9 strain might be promising for enhanced surfactin production outcome. Overall, these results emphasize the importance of strain-specific medium optimization for maximizing biosurfactant yields and productivity in industrial applications.

**Figure 3:**
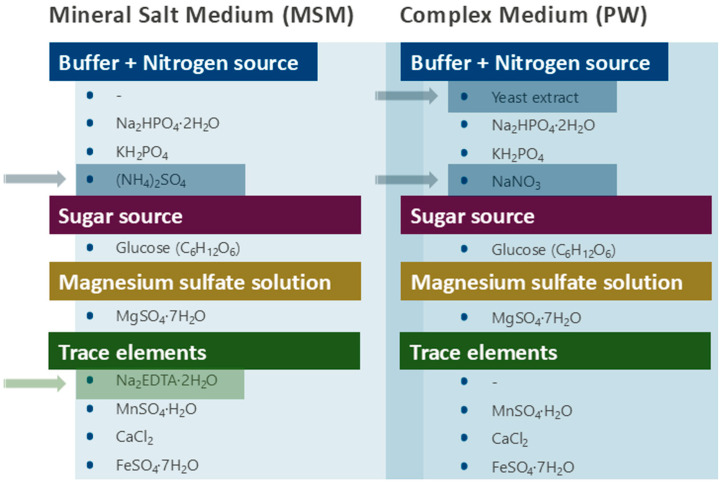
Composition of the culture media used in this study. Highlighted components and arrows indicate the differences between the two media. The first column corresponds to the minimal medium and the second to the complex medium.

The comparison between the components of the culture media used is shown in Figure 3.

It is noteworthy the exact composition of any culture medium, in particular the carbon and nitrogen sources, including their ratio. In this sense, the MSM culture medium was composed of a single defined carbon source, namely glucose (180 g/mol, containing 72 g of carbon), corresponding to 8 g of carbon/L in the medium. Regarding nitrogen, MSM contained only one defined source, ammonium sulfate ((NH_4_)_2_SO_4_), in which 28 g of nitrogen are present in 132.14 g. At the concentration used, this corresponds to approximately 0.56 g of nitrogen/L. Thus, the MSM culture medium contained 8 g/L of carbon and 0.56 g/L of nitrogen, resulting in a C/N ratio of about 14.

In contrast, the PW medium provided nitrate (NaNO_3_) and yeast extract as nitrogen sources, the latter rendering it a complex medium. The amino acids and peptides derived from the protein fraction of yeast extract can also serve as carbon sources via conversion into Krebs cycle intermediates (e.g., through α-ketoglutarate). Therefore, calculating an accurate carbon-to-nitrogen (C/N) ratio in PW is not feasible, and even an estimation could be misleading. Nevertheless, while MSM offers a defined composition and a measurable C/N ratio, PW provides a nutrient-rich and more complex environment that generally enhances microbial growth and can favor surfactin production. Previous studies report different optimal C/N ratios for surfactin biosynthesis: Ref. [24] indicated a C/N ratio of 3, whereas [25] observed maximum production at a ratio of 10, the same as that estimated for MSM.

Regarding the comparison between the two media, the two strains present similar behavior for the highest cellular concentration (Figure 2), except to *B. subtilis* BsB6 in PW.

It is expected that PW fermentations reach higher cellular concentrations, when compared to MSM, however, the exponential phases were more defined and sharper for MSM, when compared to PW, which might be due to the metabolism related to PW (glycolysis and Krebs cycle, simultaneously), and/or a misbalance between C/N sources. Thus, ideally, the culture medium should be carefully investigated, in order to be personalized to a specific strain. In addition, it should be well-outlined in terms to avoid any source of protein and amino acids; and to specific carbon and nitrogen sources.

It is fundamental to integrate the entire biotechnological process, in other words, production, purification, and application. Many studies have explored low-cost culture media for biosurfactant production, particularly using agro-industrial residues. For instance, ref. [37] highlighted cassava wastewater as a nutrient-rich, widely available, and season-independent substrate. However, the protein content in such complex substrates can hinder surfactin purification by ultrafiltration, likely in a manner similar to PW.

#### LC-MS/MS Profiling and Relative Quantification of Surfactin and Fengycin Isoforms

**Figure 4:**
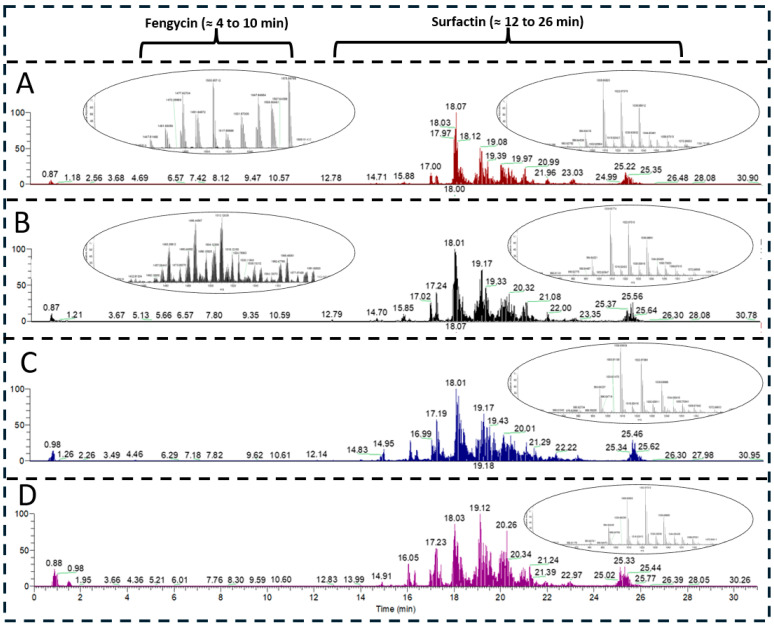
LC-MS/MS chromatographic profiles of *B. subtilis* strains cultivated in MSM (Mineral Salt Medium) ((**A**)—BMV9, brown, and (**B**)—BsB6, blue), and PW (Power Medium) ((**C**)—BMV9, black and (**D**)—BsB6, purple) media. The mass spectra of protonated fengycin and surfactin lipopeptides [M + H]^+^ eluted in the time intervals of 4–10 min and 12–26 min are shown as insets. Note that the MS spectra show the sum of all fengycin and surfactin variants within the respective time interval. Surfactin isoforms were clearly detected in all samples, whereas fengycin isoforms were only detected at very low intensities in PW medium. No fengycin signals were detected in MSM medium by LC-MS/MS, despite indications of production from alternative analytical methods.

LC-MS/MS analyses demonstrated that both *B. subtilis* strains primarily produced surfactin isoforms under both minimal and complex culture conditions. Although fengycin production was indicated by alternative analytical methods, fengycin isoforms were only detected by LC-MS/MS in complex medium and at intensities close to the detection limit. No fengycin signals were observed by LC-MS/MS in minimal medium for either strain (Figure 4). Previously, culture medium adaptation was performed for fixing this issue [19]. Accordingly, medium components need to be carefully chosen for the targeted bioproduction.

Identification of fengycin and surfactin isoforms was based on accurate mass-to-charge ratios (*m*/*z*), diagnostic MS/MS fragment ions, and chromatographic retention times. Due to overlapping fragmentation patterns, some isoforms were tentatively assigned using retention times and literature references (Table 2).

**Figure 5:**
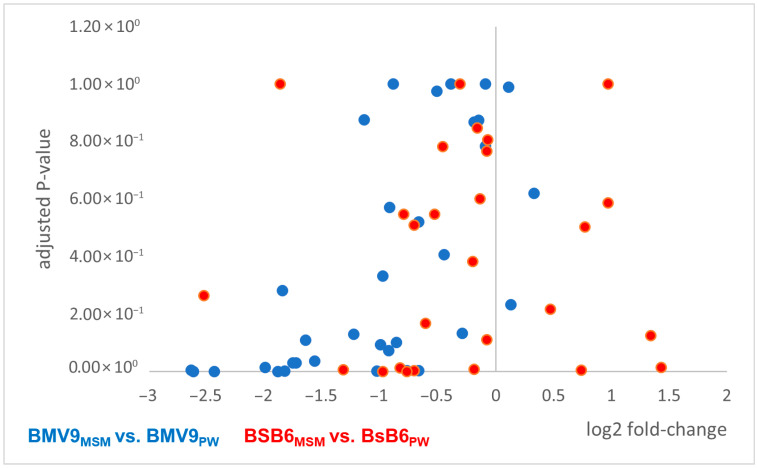
Comparative analysis of surfactin isoform abundances in *B. subtilis* strains BMV9 and BsB6 cultivated in MSM (Mineral Salt Medium) and PW (Power Medium) media. Blue dots represent the comparison between *B. subtilis* BMV9 grown in MSM and PW media (BMV9_MSM_ vs. BMV9_PW_), whereas red dots indicate the comparison between *B. subtilis* BsB6 grown under the same conditions (BsB6_MSM_ vs. BsB6_PW_).

Relative quantification revealed distinct surfactin isoform production profiles between strains and media (Supplemental Table S1). In this context, the surfactin isoforms with C14 chain lengths and Val7- or AME-variations in the amino acid part, were the most dominant version produced in both *B. subtilis* strains BMV9 and BsB6. The same trend was observed for both media (MSM and PW). In contrast, surfactin isoforms with C17 chain lengths were the least prominent versions produced. However, a previous study has shown that the longer the chain lengths of lipopeptides are, the more efficient is the antimicrobial activity due to pore formation [38]. In order to identify any effects on the production of the differently active surfactin isoforms due to the complexity of the culture media used, comparisons of isoform signal intensities were performed (Figure 5). In this context, a general reducing effect on the productivity of the *B. subtilis* BMV9 strain could be shown, while a more diverse outcome regarding the productivity was found for the *B. subtilis* strain BsB6.

However, the only slight improvements in productivity was shown in the BMV9 strain in terms of the C17 surfactin isoforms (log2 fold change of 0.13 and 0.33) when using the less complex MSM medium. In contrast, less production was found for the isoforms C13-surfactin [Val7] (−2.44) and C15-surfactin [Val7] (−2.64). In terms of the *B. subtilis* strain BsB6, the less complex MSM allowed a higher production of the isoform C17-surfactin (1.91) and a reduced abundance of C13-surfactin [Val7] (−2.53). Accordingly, both strains showed similar profiles in their biosynthetic adaptions, indicating a strain-independent mechanism for fine-tuning the abundance of surfactin isoforms in accordance with the complexity of environmental nutrients. More details regarding the MS-based quantitative surfactin measurements are provided in the Supplementary Material Table S1 (excel file).

### 3.2. Antifungal Assay

*Diaporthe* species are the causal agents of soybean seed decay and stem canker, which affect seed quality and quantity, leading to considerable economic losses. They are particularly problematic in warm and humid regions, such as the United States (especially the Midwest and Southern states), South America (notably Brazil and Argentina), and parts of Asia—key regions for global soybean production [39,40]. Since the antimicrobial properties of biosurfactants are well-documented [8,41], we tested the produced compounds against two *Diaporthe* species.

**Figure 6:**
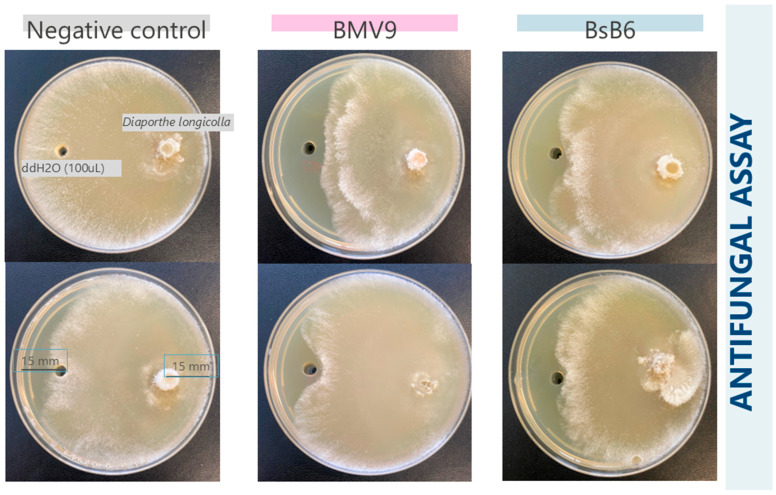
Representative plates of the antifungal assay using the 24 h cell-free supernatant of *B. subtilis* BMV9 and BsB6 cultivated in PW (Power Medium) medium against *D. longicolla*. Distilled water was used as a negative control. Each plate contains a 0.6 cm mycelial plug placed on one side and 100 µL of the test solution applied to a well on the opposite side. The photograph was taken 7 days after incubation. Inhibition zones indicate antifungal activity.

To characterize the antifungal properties of the studied strains, two inhibition assays were conducted against agriculturally relevant soybean pathogens. The results of the first assay, which utilized a 24 h supernatant against *D. longicolla*, are presented in Figure 6.

**Figure 7:**
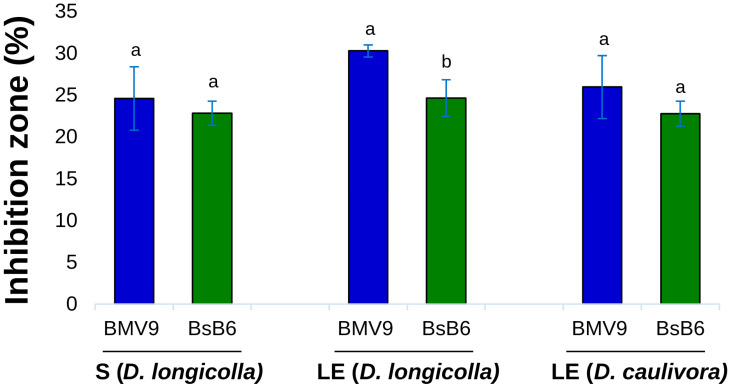
Comparison of antifungal activity exhibited by the BMV9 and BsB6 strains against the studied pathogens. The first assay (S) refers to the 24 h cell-free supernatant tested against *D. longicolla*, while the second assay (LE) corresponds to the 28 h lipopeptide extract tested against both *D. longicolla* and *D. caulivora*. All experiments were performed in triplicate, and error bars represent the standard deviation of the inhibition zone measurements. For each treatment group, data were analyzed independently by one-way ANOVA, with statistical significance determined by the F-test (*p* < 0.05).

The percentage of inhibition calculated using the PIRG formula is presented in Figure 7. The results displayed in the graph correspond to fermentation samples in PW medium, while samples in MSM medium did not produce inhibition zones when compared to the negative control. The BMV9 strain exhibited the highest inhibition zones against both *Diaporthe* species, achieving a 30.3% inhibition compared to the control group with the 28 h lipopeptide extract against *D. longicolla*. In the same assay, the BsB6 strain demonstrated a 24.6% inhibition. These differences were evaluated by one-way ANOVA (F-test), which indicated a statistically significant effect of the treatments (*p* < 0.05). Despite these differences, both strains exhibited significant inhibition rates against the pathogens, highlighting their potential for further investigation in agricultural applications.

Soybean seed decay, primarily caused by *D. longicolla*, is a major seedborne disease that significantly reduces seed quality and yield. While conventional management strategies, such as crop rotation and fungicide seed treatments, remain important, biological control offers a sustainable and environment friendly alternative. The results of this study indicate that *B. subtilis* “BMV9 and BsB6” are capable of producing lipopeptides with antifungal properties. The antifungal activity observed was statistically supported by one-way ANOVA (*p* < 0.05). Therefore, further in vitro and in vivo studies (under greenhouse and field conditions) are essential to evaluate their effectiveness and practical application in integrated disease management.

### 3.3. Oil Displacement Test

**Figure 8:**
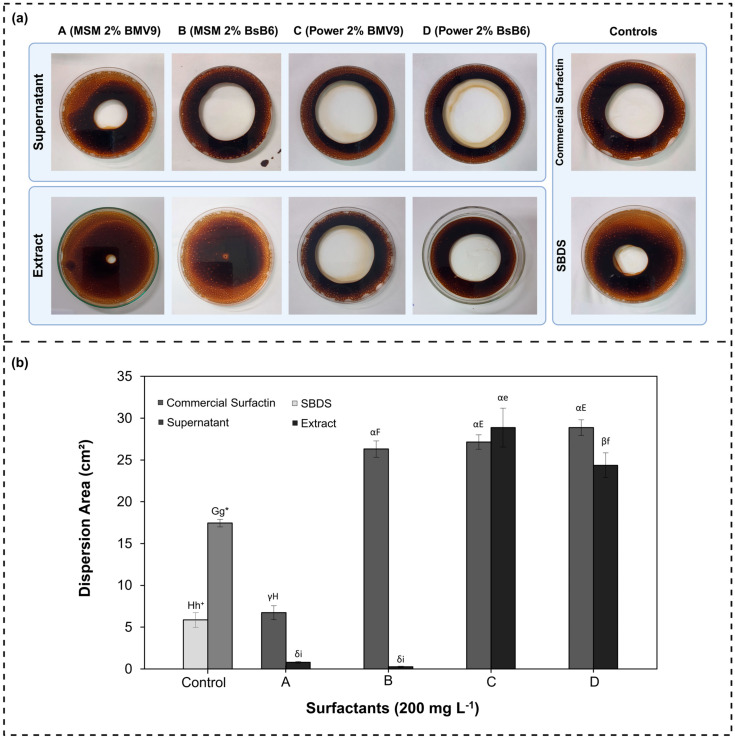
(**a**) Representative images of the oil displacement test using surfactin produced by BMV9 and BsB6 cultivated in MSM and PW media, applied as supernatant or extract (200 mg·L^−1^); larger halos indicate higher oil displacement efficiency. (**b**) Oil displacement area (cm^2^) for samples A (BMV9–MSM), B (BMV9–PW), C (BsB6–MSM), and D (BsB6–PW), evaluated as supernatant and extract surfactin, and compared with commercial surfactin and SBDS controls. Two-way ANOVA (sample × supernatant/extract) followed by Tukey’s HSD is represented by different Greek letters. One-way ANOVA followed by Tukey’s HSD comparing supernatants is indicated by uppercase letters, whereas comparisons among extracts are indicated by lowercase letters. Symbols (*, ^+^) denote significant differences between commercial surfactant controls (*p* < 0.05).

Oil displacement tests were conducted using solutions at 200 mg/L, derived from the supernatant and the lipopeptide extract (24 h fermentation samples). Their performance was compared with commercial surfactin and the synthetic surfactant SDBS. The visual oil dispersion areas are shown in Figure 8a and their numerical areas are shown in Figure 8b. It is worth mentioning that the larger the area, the greater the surfactant’s potential to be used in EOR and other stages of the petrochemical industry.

In the control group, which included the commercial surfactants (surfactin and SDBS), commercial surfactin resulted in approximately 23% greater oil displacement compared to SDBS. Within the fermentation-derived solutions, samples B, C, and D demonstrated results compared to the commercial surfactants, (supernatants) as illustrated in Figure 8a,b. Statistical analysis by one-way ANOVA followed by Tukey’s HSD confirmed significant differences between the commercial surfactants and the fermentation-derived supernatants (*p* < 0.05).

Comparing the two groups of produced surfactin, the oil displacement area promoted by the surfactin in the supernatant was greater than that obtained from the extract. For samples cultivated in MSM medium, the displacement area was 11.81% (sample A) and 51.85% (sample B) higher in the supernatant. In contrast, for samples produced in PW medium, the differences were less than 9% (Figure 8b), confirmed by two-way ANOVA (sample × supernatant/extract), which indicated a significant effect of non-purification step, with supernatants showing higher oil displacement than extracts (*p* < 0.05). Ref. [6] reported bigger oil dispersion areas using purified surfactin obtained through acid precipitation, reaching approximately 40 cm^2^. However, acid precipitation is a non-selective process and may result in the co-precipitation of other compounds that could also contribute to oil displacement.

Samples A to D were obtained from different fermentation processes. However, it was observed that the PW medium, used for samples C and D, resulted in bigger oil displacement areas ranging from 24.36 to 28.87 cm^2^. Statistical comparison indicated that samples produced in PW medium were significantly superior to those obtained in MSM medium (*p* < 0.05). These results should be correlated to surfactin content (similar to all of them, A to D), and also to other surface activity compounds such as fengycin, proteins, synergism among them, etc. Thus, it should take into account that the culture medium can affect the yield of surfactin (total), the isoforms, and also the production of other biosurfactants.

The composition of the culture medium directly influences the yield of biosurfactant production and its physicochemical properties [42]. Therefore, the use of the complex PW medium may have enhanced the system’s capacity to reduce surface tension, leading to more efficient oil displacement [6,37,43].

For petrochemical applications such as microbial enhanced oil recovery (MEOR), surfactin samples produced using the complex PW medium are more promising than those obtained with the MSM medium (the surfactin concentration of 48 h samples were significantly higher for PW than MSM). Notably, purification processes represent approximately 70–80% of the total production costs in bioprocess [44,45]. Therefore, the direct application of surfactin present in the supernatant offers high potential for MEOR, as it may reduce overall process costs by eliminating expensive purification steps. This approach is economically attractive, as the crude surfactant does not require further refinement, making its use more cost-effective compared to highly purified biosurfactants and conventional synthetic surfactants.

## 4. Conclusions

In this study, two genetically modified *B. subtilis* strains, BMV9 and BsB6, were comparatively evaluated for their growth performance and lipopeptide production under minimal (MSM) and complex (PW) culture media. The results demonstrated that both strains were capable of producing surfactin in both media, while fengycin was primarily detected in PW medium. BMV9 consistently outperformed the BsB6 strain in terms of biomass yield, surfactin and fengycin titers, especially under nutrient-rich conditions, confirming its enhanced biosynthetic potential.

The antifungal assays revealed that both supernatants and lipopeptide extracts exhibited inhibitory activity against *Diaporthe* spp., with BMV9 showing the strongest effects. Additionally, biosurfactant solutions derived from PW medium demonstrated superior oil displacement capacity, particularly when using the supernatant directly, highlighting the feasibility of using minimally processed biosurfactants for petrochemical applications.

PW is likely a more feasible culture medium, when compared to MSM, for lipopeptide production, followed by the petrochemical industrial application. The results are better than synthetic surfactants, and they also represent a much more sustainable approach and have less impact on the environment.

These findings highlight the importance of selecting appropriate strain–medium combinations to optimize biosurfactant production. However, the current yields remain low, reinforcing the need to further enhance production for future industrial applications. The demonstrated use of crude culture supernatants in functional applications, such as biocontrol and oil dispersion, also underscores the strong biotechnological potential of these strains for sustainable and cost-effective processes. Building on existing studies, particularly those showing the effectiveness of cultivation strategies like fed-batch fermentation to increase surfactin yields, future work should focus on adapting these approaches to high-producing strains. This includes integrating agro-industrial residues as sustainable substrates and evaluating downstream processes compatible with crude supernatants. Together, these strategies will be essential to close the gap between laboratory-scale production and the industrial deployment of surfactin-based systems.

## Figures and Tables

**Table 1:** Comparison among media in terms of the specific growth rate (μ), yield biomass/substrate (Y_X/S_), yield product/substrate (Y_P/S_), yield product/biomass (Y_P/X_) and specific productivity product/biomass (q_P/X_). MSM (Mineral Salt Medium) and PW (Power Medium).

Strain	Medium			Surfactin			Fengycin		
		μ (h^−1^)	Y_X/S_ (g/g)	Y_P/S_ (g/g)	Y_P/X_ (g/g)	q_P/X_ (g/g·h)	Y_P/S_ (mg/g)	Y_P/X_ (mg/g)	q_P/X_ (mg/g·h)
BMV9	MSM	0.172	0.202	0.075	0.482	0.027	0.173	1.114	0.062
	PW	0.285 *	0.205 *	0.078 *	5.284 *	0.110 *	0.203 *	13.67 *	0.285 *
BsB6	MSM	0.094	0.182	0.085	0.707	0.039	0.057	0.335	0.010
	PW	0.212 *	0.078 *	0.052 *	1.491 *	0.031 *	0.141 *	4.817 *	0.172 *

* Theoretical values presented are based on the added glucose as the sole quantified carbon input. Contributions from other, unmeasured carbon sources within the complex medium may lead to deviations from these theoretical estimates.

**Table 2:** The most intense spectra surfactin homologs: *m*/*z* and possible amino acid sequence variations. MSM (Mineral Salt Medium) and PW (Power Medium).

*m*/*z*	BMV9-MSM	BMV9-PW	BsB6-MSM	BsB6-PW	Ref
≈994	*^(4)^ BSACS ^†^-C_12_; and/or C_12_[Val_2_]; and/or C_13_[Val_7_]	*^(4)^ BSACS ^†^-C_12_; and/or C_12_[Val_2_]; and/or C_13_[Val_7_]	*^(4)^ BSACS ^†^-C_12_; and/or C_12_[Val_2_]; and/or C_13_[Val_7_]	*^(4)^ BSACS ^†^-C_12_; and/or C_12_[Val_2_]; and/or C_13_[Val_7_]	[29]
≈1008	*^(1)^ BSACS ^†^-C_13_; and/or C_13_[Val_7_]; and/or C_13_[Val_2_]; and/or C_13_[Val_7_]	*^(1)^ BSACS ^†^-C_13_; and/or C_13_[Val_7_]; and/or C_13_[Val_2_]; and/or C_13_[Val_7_]	*^(1)^ BSACS ^†^-C_13_; and/or C_13_[Val_7_]; and/or C_13_[Val_2_]; and/or C_13_[Val_7_]	*^(2)^ BSACS ^†^-C_13_; and/or C_13_[Val_7_]; and/or C_13_[Val_2_]; and/or C_13_[Val_7_]	[29]
≈1022	*^(2)^ BSACS ^†^-C_14_; and/or C_14_[Val_2_]; and/or C_14_[Val_7_]; and/or C_14_[AME] ^††^	*^(2)^ BSACS ^†^-C_14_; and/or C_14_[Val_2_]; and/or C_14_[Val_7_]; and/or C_14_[AME] ^††^	*^(2)^ BSACS ^†^-C_14_; and/or C_14_[Val_2_]; and/or C_14_ [Val_7_]; and/or C_14_[AME]^††^	*^(1)^ BSACS ^†^-C_14_; and/or C_14_[Val_2_]; and/or C_14_[Val_7_]; and/or C_14_[AME] ^††^	[29]
≈1036	*^(3)^ C_15_[AME] ^††^; C_15_[Val_2_]; C_15_[Val_7_]	*^(3)^ C_15_[AME] ^††^; C_15_[Val_2_]; C_15_[Val_7_]	*^(3)^ C_15_[AME] ^††^; C_15_[Val_2_]; C_15_[Val_7_]	*^(3)^ C_15_[AME] ^††^; C_15_[Val_2_]; C_15_[Val_7_]	[29]
≈1044	*^(5)^ BSACS ^†^-C_14_	*^(5)^ BSACS ^†^-C_14_	*^(5)^ BSACS ^†^-C_14_	*^(5)^ BSACS ^†^-C_14_	[30]
≈1058	*^(6)^ BSACS ^†^-C_15_; **C_15_[GME+Val_7_]	*^(6)^ BSACS ^†^-C_15_; **C_15_[GME+Val_7_]	*^(6)^ BSACS ^†^-C_15_; **C_15_[GME+Val_7_]	*^(6)^ BSACS ^†^-C_15_; **C_15_[GME+Val_7_]	[29,30]
≈1072	*^(7)^ BSACS ^†^-C_16_; **C_15_[GME+Ile_7_]	*^(7)^ BSACS ^†^-C_16_; **C_15_[GME+Ile_7_	*^(7)^ BSACS ^†^-C_16_; **C_15_[GME+Ile_7_	*^(7)^ BSACS ^†^-C_16_; **C_15_[GME+Ile_7_	[29,30]
≈1086	*^(8)^ BSACS ^†^-C_17_	*^(8)^ BSACS ^†^-C_17_	*^(8)^ BSACS ^†^-C_17_	-	[30]
**Other homologs**					
≈1030	-	-	BACS ^†^-C_13_ with precursor ion [M + Na]^+^	BACS ^†^-C_13_ with precursor ion [M + Na]^+^	[30]
**Fengycin**					
≈1477	*^(2)^ BFACS ***-C_17_ [M + H]^+^	*^(3)^ BFACS ***-C_17_ [M + H]^+^	-	-	[31]
≈1491	*^(3)^ [Val_1_]-C_16_ [M + H]^+^	*^(1)^ [Val_1_]-C_16_ [M + H]^+^	-	-	[31]
≈1505	*^(1)^ [Val_1_]-C_17_ [M + H]^+^	*^(2)^ [Val_1_]-C_17_ [M + H]^+^	-	-	[31]

*^(n)^ The numerical ordering (column) according to the most intensity of the spectrum (e.g., *^(1)^ the highest spectrum). ^†^ The base of surfactin amino acid sequence—BSACS (Glu-Leu-Leu-Val-Asp-Leu-Leu)-C_13_,-C_14_,-C_15_, -C_16_, or -C_17_. ** GME—glutamic acid 5-methyl ester. ^††^ AME—aspartic acid 4-methyl ester. *** The base of fengycin amino acid sequence—BFACS (Ala-Ile-Pro-allo-Thr(3)-Glx-Tyr-Tyr-Orn).

## Data Availability

The original contributions presented in this study are included in the article/Supplementary Materials. Further inquiries can be directed to the corresponding authors.

## Supplementary Materials

The following supporting information can be downloaded at https://www.mdpi.com/article/10.3390/biology15020146/s1. Table S1: Comparisons of isoform signal intensities (e.g., sample A vs. C—the *B. subtilis* BMV9 in MSM or PW, respectively; and sample B vs. D—the *B. subtilis* BsB6 in MSM or PW). Reference [29] is cited in the Supplementary Materials.
